# “Would you want to know?” Questions of utility and responsibility in Italian laypersons’ preferences about genetic risk communication

**DOI:** 10.1007/s12687-025-00837-9

**Published:** 2025-11-18

**Authors:** Lea Godino, Daniela Turchetti, Linda Battistuzzi, Liliana Varesco, Elena Nardi, Vanessa Gentili, Paolo Chiari, Alvisa Palese

**Affiliations:** 1 Medical Genetics Unit, IRCCS Azienda Ospedaliero-Universitaria Di Bologna, Bologna, Italy; 2 Department of Biomedicine and Prevention, University of Rome “Tor Vergata”, Viale Montpellier, 1- 00128 Italy, Rome Italy; 3 Department of Medical and Surgical Sciences, University of Bologna, Bologna, Italy; 4 Medical Oncology Unit 2, IRCCS Ospedale Policlinico San Martino, Genoa, Italy; 5 Unit of Hereditary Cancer, IRCCS Ospedale Policlinico San Martino, Genoa, Italy; 6 Research and Innovation Unit, IRCCS Azienda Ospedaliero, Universitaria Di Bologna, Bologna, Italy; 7 Department of Medicine, University of Udine, Udine, Italy

**Keywords:** Cross-sectional, Public, Genetic risk, Genetic information, Moral responsibility

## Abstract

We surveyed the preferences of Italian laypersons (*n* = 609) regarding the communication of genetic risk information to and within families, presenting scenarios about three genetic conditions (Cystic Fibrosis, CF; Hereditary Cancer, HC; and early-onset Alzheimer’s disease, AD). We found that almost all of the participants (565/609, 93%) were interested in receiving genetic risk information about at least one of the diseases, and 95% of these also stated they would have genetic testing. Interestingly, 78% of participants in this subset wanted to be informed of their genetic risk and have testing for all the genetic conditions presented in the survey. The main motivating factors were prevention for HC, reproductive responsibility for CF, and life-planning and emotional preparedness for AD, covering a range of elements of clinical, health-related and personal utility. In addition, over a third of our study participants (198/565, 35.0%) indicated that both family members and healthcare professionals are morally responsible for communicating genetic risk information; over a fourth (148/565 26.2%) felt that the responsibility lies with everyone involved: family members, healthcare professionals and also themselves. Findings indicate that bringing into focus individual needs that go beyond medical care may help improve communication about genetic risk and informed choices about genetic testing. Moreover, the Italian public opinion may be open to the possibility of a proactive role for healthcare professionals in disseminating genetic risk information to family members, particularly within the frame of a collaborative effort.

**Supplementary Information:**

The online version contains supplementary material available at 10.1007/s12687-025-00837-9.

## Introduction

Advances in genetic testing have significantly improved the ability to identify patients affected by hereditary diseases (Nherera et al. 2011; Godino et al. 2016, 2018, 2019; Wassall et al. 2017; Cortesi et al. 2020; Bertonazzi et al. 2022; Sessa et al. 2023). For these patients, undergoing genetic testing and learning that they carry a pathogenic variant (PV) can have substantial benefits, including targeted treatment options, preventive measures, informed reproductive decisions, and long-term life planning (Bos et al. 2007; Nherera et al. 2011; Razzaboni et al. 2012; Wassall et al. 2017; Cortesi et al. 2020; Bertonazzi et al. 2022; Sessa et al. 2023; Godino et al. 2025a; Fontoura Dias et al. 2025). It also provides an opportunity to determine, through an inexpensive and specific test, which other family members have inherited the PV and which have not, in a process of sequential genetic counselling and testing that is termed cascade genetic testing (Frey et al. 2022).

The specific value of learning about carrying a PV depends on the disease and is not always easy to define in terms of its key components, such as clinical and personal utility involving psychological, social and practical benefits(Bunnik et al. 2015). For instance, strong clinical benefits are widely recognized for many cancer predisposition syndromes (Razzaboni et al. 2012; Cortesi et al. 2020; Bertonazzi et al. 2022), whereas medical advantages are more limited for recessive diseases, in which patients may undergo testing mostly for family planning and risk assessment (Cannon et al. 2019), or for dominant forms of degenerative diseases like Alzheimer’s (Jakobsen et al. 1999; Watts et al. 2012).

In routine genetic counselling practice, the responsibility to share information about genetic risk with family members is placed on the proband, the individual whose genetic test first revealed the PV. In this model, the genetics professional encourages the proband to disclose the genetic risk information to their at-risk relatives, so they, in turn, have the opportunity to seek genetics services (Samuel et al. 2017; Frey et al. 2022; Grill et al. 2024; Fontoura Dias et al. 2025). Italian practice follows the same logic: once the proband has been identified, that person is expected to alert at‑risk family members, with healthcare professionals playing a supportive role at the most (Varesco et al. 2025). This proband‑mediated approach is endorsed by both national Italian and international guidelines, all of which highlight that communicating with relatives is chiefly the patient’s responsibility (Dutch Society for Clinical Genetics, 2019; Migliara et al. 2017).

Even when scientific evidence strongly supports testing, however, probands may find the experience of informing their family of their genetic diagnosis and its implications burdensome (Shin et al. 2021; Hawranek et al. 2021; Nevin et al. 2022; Tran et al. 2025; Costanzo et al. 2025). Beyond the challenge of understanding the potential health benefits that cascade testing can provide to relatives, psychological and relational factors may create additional difficulties (Kne et al. 2017; Di Pietro et al. 2020; Hawranek et al. 2021; Murphy et al. 2024; Fontoura Dias et al. 2025) Probands often describe conversations with at‑risk relatives as psychologically onerous, experience anxiety or guilt about disclosing their own results, harbour privacy concerns, and fear discrimination in personal or professional spheres (Srinivasan et al. 2020). They may also worry about relatives’ reactions and about upsetting them with unwelcome news (Afaya et al. 2024). Interpersonal conflict, emotional distance, or estrangement within the family can also complicate the communication of genetic risk information, as can low health literacy (Williams et al. 2019) and socio‑economic constraints, particularly those affecting medically underserved or otherwise vulnerable groups (Ahsan et al. 2023). As a result, the proband-mediated genetic information model is often ineffective, leading to suboptimal uptake of cascade counselling and testing (O’Reilly et al. 2008; Frey et al. 2022; Trevisan et al. 2023; Caeser et al. 2024).

In recent years and for certain diseases, this has prompted a growing interest in exploring issues regarding moral responsibilities associated with disseminating genetic risk information to at-risk relatives (Dheensa et al. 2016b; Grill and Rosén 2020; Aceti et al. 2024), as well as the possibility of healthcare professionals (HCPs) taking on an active role in this communicative process. This has led to the introduction of a model in which HCPs directly inform family members, known as direct contact (Frey et al. 2022). Although the most effective, equitable, and sustainable strategy for its implementation remains unclear, the direct contact approach may increase family members’ engagement. Moreover, it aligns with research showing that HCPs often feel a professional obligation to inform family members at risk (Stol et al. 2010). However, HCPs may also perceive that being directly involved in disclosing genetic risk information to family members may be ethically problematic or uncalled for (Stol et al. 2009; Grill and Rosén 2020; Grill et al. 2024), or that it would place an excessive strain on limited healthcare resources. In addition, legal frameworks differ across countries: for instance, Australian legislation allows HCPs to directly contact at-risk relatives without breaching privacy regulations (Henrikson et al. 2020; Tiller et al. 2023a), whereas in France, probands are legally required to inform their relatives or authorize physicians to do so anonymously (Law No. 2021–1017 dated 2 August 2011 (art. 15) 2021).

Public expectations and preferences can help guide HCPs in choosing appropriate approaches to genetic risk communication, as societal attitudes inevitably influence the acceptance and effectiveness of different strategies, particularly because members of the public could be unaffected at-risk relatives of probands. Moreover, as genetic testing becomes more broadly used across various medical settings and is increasingly accessible to consumers, the views of laypersons have grown more significant. Therefore, to explore this topic in the Italian context, this study investigated laypersons’ preferences regarding how genetic risk information should be communicated to and within families, focusing on three genetic conditions (Cystic Fibrosis, Hereditary Cancer, and early-onset Alzheimer’s disease), which differ in clinical utility and implications. Particular attention is given to perceived responsibilities and preferred communicative roles.

## Materials and methods

### Design

A cross-sectional design was conducted, here reported according to the STrengthening the Reporting of OBservational Studies in Epidemiology (STROBE).

### Sample and setting

Adults' participants from the Italian general population were recruited through various digital channels such as Facebook© (personal pages and interest groups), Instagram©, LinkedIn©, and personal mailing lists of the authors and their network. Data collection was conducted using an anonymous questionnaire hosted on the Microsoft Forms platform. The survey remained open to respondents from November 24th, 2024, to February 7th, 2025.

An individual could participate if they: (1) were over 18 years of age, (2) had no personal or family history of hereditary disease, (3) were able to give informed consent, and (4) were able to speak Italian fluently. For the purpose of this study, HCPs were excluded.

### Questionnaire

The questionnaire was designed for the purposes of this research. Given its ad hoc nature, a pilot phase was conducted to assess its comprehensibility and ensure that the questions were clear and appropriately framed. Based on feedback from this initial phase, several modifications were made before administering the questionnaire on a larger scale. Further details regarding the development and refinement process of the questionnaire can be found in a previous publication (Godino et al., 2025).

The questionnaire was designed to assess the general population's literacy, perceptions, and attitudes toward genetic conditions, genetic testing, and the communication of genetic risk within families. It comprises eight sections and includes both closed-ended (dichotomous, multiple choice, and 5-point Likert scales) and open-ended questions. A conditional logic structure was implemented to tailor the response flow based on participants’ answers. The structure and the summary of the questionnaire are shown in Table 1, and its complete version is reported in the Supplementary File 1.

**Table 1:** Structure and summary of the Questionnaire

Section	Item	Summary
1. Genetic Conditions and Genetic Testing	1–2	This section assesses participants’ general awareness and understanding of genetic testing. Respondents were asked whether they had ever heard or read about genetic testing. If they answered affirmatively, they were then asked to select which purposes they associated with genetic testing (i.e., assessing disease risk, guiding treatment, predicting drug response, evaluating reproductive risk)
2. Personal and Family Experience	3–6	Participants were then asked whether they were aware of any hereditary conditions in their family (yes/no) and to specify which conditions. Those who answered “no” or were unsure were automatically directed to the next section
3. Hypothetical Scenarios of Hereditary Diseases	7–12	This section presented three hypothetical clinical scenarios involving a relative diagnosed with a one of the following hereditary conditions: cystic fibrosis, hereditary breast and ovarian cancer, and early-onset Alzheimer’s disease. In each scenario, respondents were informed that they had a 25% probability (1 in 4) of having inherited the same genetic variant identified in their relative. After reading all three scenarios, participants were asked a general question: “Would you want to know?”. If they answered “no,” they were automatically directed to Sect. 7. If they answered “yes,” they were subsequently asked to specify for which of the three conditions they would want to be informed, and to explain their motivations. Participants were also asked whether they would be willing to undergo genetic testing and invited to explain their reasoning
4. Moral Responsibility	13	Participants who stated they would want to know were asked who they believed held the moral responsibility to inform them. A brief introduction clarified that, although healthcare professionals usually recommend that affected individuals inform their relatives, this communication can be difficult and often does not occur. Participants indicated whether they believed the responsibility lay with themselves, their family members, or healthcare professionals, responding “Yes,” “No,” or “I don’t know” to each. An open-ended option allowed them to specify other figures
5. Communication Preferences	14–25	This section explored participants’ preferences regarding both the source and the mode of communication of the risk of having inherited the family variant. Respondents indicated their level of agreement (5-point Likert scale) with the statement “I would prefer to be informed by a family member” and “I would prefer to be informed by a health care professional”. Those who selected “Agree” or “Strongly agree” were then asked to specify which kind of relative/health care professional they would prefer to receive the information from, and how they would prefer this communication to occur Then, all respondents rated their agreement (5-point Likert scale) with four specific statements regarding communication preferences (e.g., “I would prefer to be informed by a family member with whom I have no contact rather than not being informed at all”)
6. Disclosure of Personal Diagnosis	26–31	This section investigated how participants would approach the communication of genetic risk if they were if they were the family’s informant. Participants were asked to choose which family members should be informed among closed options. Those who indicated that none should be informed were directed to an open-ended question to explain their reasons. Other participants rated their agreement (5-point Likert scale) with three communication strategies: informing relatives personally, delegating the task to healthcare professionals, doing so with professional support
7. Family Relationships	32–33	This section evaluates respondents' perceptions of their family's communication practices using the Italian version of SCORE-15 (Systemic Clinical Outcome and Routine Evaluation) (Paolini & Schepisi, 2020)
8. Socio-Demographic Characteristics		This section collects demographic information

### Variables

A continuous genetic literacy score (0–4) was calculated from four yes/no items assessing genetic literacy (Item 2), assigning one point for each correct answer. A composite variable assessed perceived moral responsibility (Item13), recoding “Yes” as 1 and both “No” and “Don’t know” as 4. The three responses (self, family, HCP) were concatenated into a string variable, and participants were grouped into eight categories: “Yes to all,” “Only self,” “Only family,” “Only HCPs,” combinations of two, or “No/Don’t know to all.” A categorical variable was derived by comparing Likert-scale responses to Item14 (preference for being informed by a family member) and Item17 (preference for being informed by an HCP): participants were classified as “Prefers family member” (Item14 > Item17), “Prefers healthcare professional” (Item17 > Item14), “Equally agrees” (Item14 = Item17 ≥ 3), and “Disagrees with both” (Item14 = Item17 < 3). A binary variable captured interest in receiving information about all three conditions (Item8): participants who answered “yes” to all scenarios were coded as 1; all other combinations were coded as 0. Another categorical variable was created by comparing Item27 (preference to inform relatives personally) and Item28 (preference for HCPs to do so): “Would inform personally” (Item27 > Item28), “Prefer professionals” (Item28 > Item27), “Equally agrees” (Item27 = Item28 ≥ 3), and “Disagrees with both” (< 3).

### Data analysis

The dataset was directly exported from Microsoft Forms. Statistical analyses were conducted using IBM-SPSS (Version 28). Descriptive statistics were reported as means, standard deviations, and ranges for continuous variables, and absolute and relative frequencies for categorical variables. Pearson’s chi-squared test was applied to examine associations between nominal variables, while Fisher’s exact test was used for dichotomous variables where appropriate. Group differences were analyzed using one-way ANOVA, with Bonferroni correction for multiple comparisons. A T-test was used for pairwise comparisons. Binary logistic regression was performed to assess the association between a binary outcome and a set of predictor variables. Statistical significance was set at two-tailed p < 0.05.

Open-ended responses were analysed using a descriptive qualitative approach, following the thematic analysis method outlined by Braun and Clarke (Braun and Clarke 2006). Key themes and illustrative data excerpts have been synthesized and are presented in tabular format to facilitate interpretation. The analysis was conducted by two researchers (LG and VG), who independently read and coded the responses to identify recurring patterns and themes. They then met to compare and discuss their interpretations. Any differences were openly debated and thoughtfully resolved in discussion with a third researcher (LB), which helped to enrich the analysis and ensure its consistency and trustworthiness.

### Ethical considerations

The study followed the principles of the Declaration of Helsinki and obtained ethics approval from the Ethics Committee of the University of Bologna (Italy) on September 30th, 2024 (approval number 0313516 of 11th October, 2024).

## Results

**Fig. 1:**
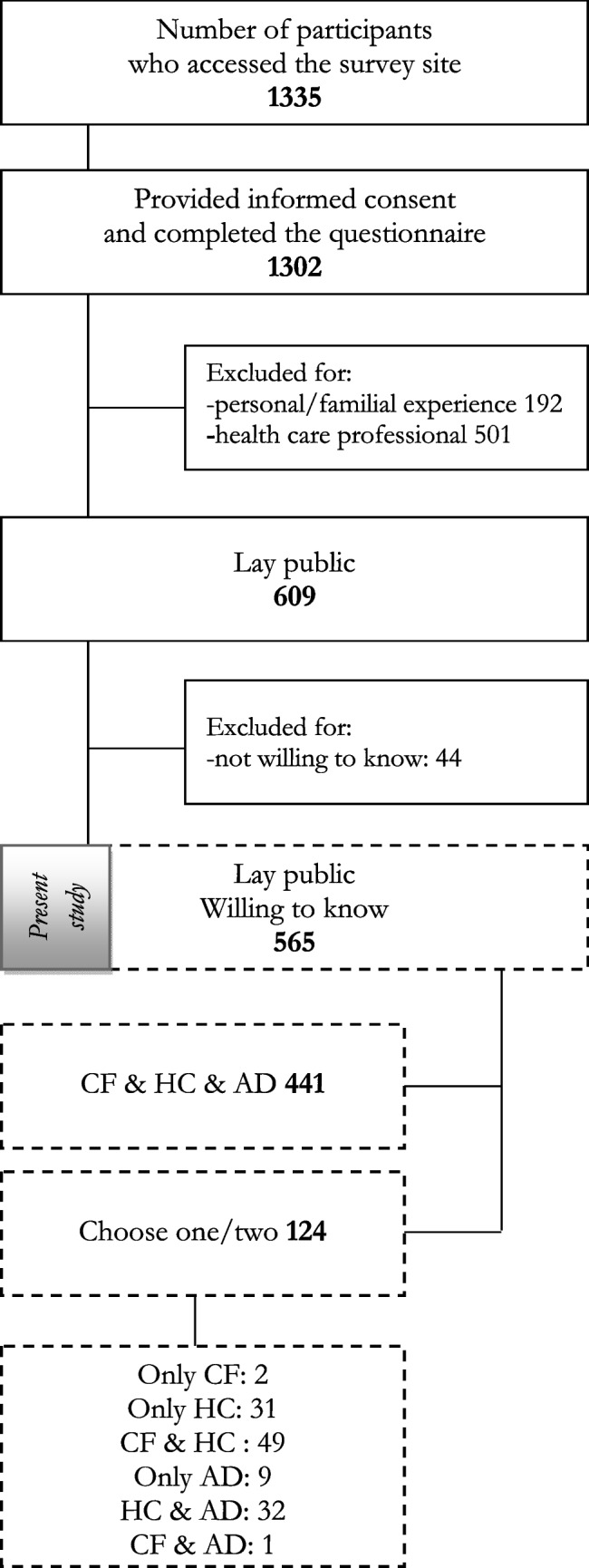
Flowchart of Participant Recruitment and Selection

The diagram of survey participants is shown in Fig. 1. Of the 1335 individuals who accessed the survey, 1302 provided consent and completed it. After excluding 192 respondents with a personal or family history of hereditary conditions and 501 HCPs, the final sample included 609 lay individuals (Table 2). The sample had a mean age of 39.8 ± 14.8 years (range: 18–79), with a majority being female (68.5%) and university-educated (48.9%). Family cohesion, assessed via the Score-15, averaged 2.05 ± 0.61 (range: 1.00–4.40), suggesting moderate relational functioning (results for single questions listed in Supplementary Table S1). Participants’ genetic literacy was low overall (1.93 ± 1.73, range 0–4): 244 of 609 (40.1%) scored 0, 198 (32.5%) scored 4, and the remainder were distributed across intermediate scores (score1: 6, 1.0%; score2: 103, 16.9%; score3: 58, 9.5%). The four items used to assess genetic literacy are detailed in Table S2.

**Table 2:** Characteristics of the study participants and comparisons of groups with different “Wanting to Know” responses

	All participants		Wanting to be informed about at least one condition versus none					Wanting to be informed about three conditions versus one or two conditions				
	(*n*=609)		None (*n*=44)		At least one (*n*=565)			1 or 2 conditions (*n*=124)		All 3 conditions (*n*=441)		
Characteristics of the study participants	Mean	SD	Mean	SD	Mean	SD	*p-value*	Mean	SD	Mean	SD	*p-value*
Age	39.75	14.78	38.61	14.51	39.84	14.81	*0.596*	42.21	15.56	39.18	14.54	*0.044*
	*n*	%	*n*	%	*n*	%		*n*	%	*n*	%	
Gender*												
Male	181	29.7	16	37.2	165	29.7	*0.305*	32	26.7	133	30.6	*0.432*
Female	417	68.5	27	6.5	390	70.3		88	73.3	302	69.4	
Marital Status												
Single	227	37.3	18	40,9	209	37,0	*0.643*	45	36.3	164	37.2	*0.017*
Married	211	34.6	14	31,8	197	34,9		51	41.1	146	33.1	
Cohabiting	144	23.6	9	20,5	135	23,9		19	15.3	116	26.3	
Divorced	21	3.4	3	6,8	18	3,2		8	6.5	10	2.3	
Widowed	6	1.0	0	0.0	6	1,1		1	0.8	5	1.1	
Children												
No	348	57.1	26	59.1	322	57	*0.875*	64	51.6	258	58.5	*0.183*
Yes	261	42.9	18	40.9	243	43		60	48.4	183	41.5	
Highest Level of Education**												
Middle School Diploma	50	8.2	6	13.6	44	7.8	*0.305*	9	7.3	35	8.0	*0.679*
High School Diploma	260	42.8	18	40.9	242	42.9		52	41.9	190	43.2	
Bachelor’s Degree	210	34.5	16	36.4	194	34.4		48	38.7	146	33.2	
Postgraduate Specialization	64	10.5	2	4.5	62	11.0		13	10.5	49	11.1	
Doctorate (PhD)	24	3.9	2	4.5	22	3.9		2	1.6	20	4.5	
Current Employment Status ***												
Paid employment	399	65.8	29	65.9	370	65.8	*0.267*	80	65.0	290	66.1	*0.743*
Volunteer	4	0.7	0	0.0	4	0.7		1	0.8	3	0.7	
Student	130	21.5	8	18.2	122	21.7		28	22.8	94	21.4	
Household responsibilities	19	3.1	4	9.1	15	2.7		5	4.1	10	2.3	
Unemployed	27	4.5	2	4.5	25	4.4		3	2.4	22	5.0	
Retired	27	4.5	1	2.3	26	4.6		6	4.9	20	4.6	
Religious Orientation												
Believer (Practing/Non-practicing)	401	65.8	32	72.7	369	65.3	*0.409*	86	69.4	283	64.2	*0.337*
Non-Believer/Agnostic	208	34.2	12	27.3	196	34.7		38	30.6	158	35.8	
Current Area of Residence****												
Northern Italy	345	56.7	18	40.9	327	57.9	*0.033*	79	63.7	248	56.2	*0.449*
Central Italy	118	19.4	8	18.2	110	19.5		19	15.3	91	20.6	
Southern Italy and Islands	136	22.3	16	11.8	120	21.2		24	19.4	96	21.8	
Abroad	10	1.6	2	4.5	8	1.4		2	1.6	6	1.4	
Genetic literacy	1.93	1.73	2.07	1.68	1.92	1.74	*0.596*	1.75	1.73	1.97	1.74	*0.208*
Family relationship (Score-15)	2.05	0.61	2.10	0.51	2.05	0.61	*0.621*	2.05	0.59	2.05	0.62	*0.975*

^*^Eleven respondents who selected 'prefer not to disclose' for sex were excluded and treated as missing data

^**^One missing value for the variable 'Highest Level of Education'

^***^Three missing values for the variable 'Current Employment Status'

^****^ A new variable was created by recoding the original region of residence variable into four geographical macro-areas: Northern Italy (Emilia-Romagna, Friuli Venezia Giulia, Liguria, Lombardy, Piedmont, Trentino-Alto Adige/South Tyrol, Aosta Valley, Veneto), Central Italy (Lazio, Marche, Tuscany, Umbria), Southern and Insular Italy (Basilicata, Calabria, Campania, Molise, Apulia, Sardinia, Sicily), and Abroad (residing outside Italy)

### Wanting to know one’s genetic risk and have genetic testing

Of the 609 participants, 565 (92.7%) stated they would want to be informed about at least one condition in the hypothetical scenarios, while 44 (7.3%) would prefer not to receive any information. Among those open to information, 441 (78.0%) stated that they would want to know in all three scenarios, and 124 (22.0%) selected one or two, most frequently Hereditary Cancer (HC, 90.3%), followed by Cystic Fibrosis (CF, 41.9%) and early-onset Alzheimer’s disease (early-onset AD, 33.8%). Detailed choice combinations are shown in Fig. 1, and display highly fragmented responses. Therefore, answers were amalgamated into two larger groups (“Wanting to be informed about one or two conditions” and “Wanting to be informed about the three conditions”). Comparisons are presented in Table 2. No significant differences were found across groups in terms of participants’ socio-demographic features, genetic literacy, or family cohesion score.

Open-ended responses to Item 9 (Why would you want to know?) offered insight into the drivers of participants’ decisions. Motivations varied by scenario: reproductive responsibility (CF), prevention (HC), and life planning and emotional preparedness (early-onset AD) (see Tables S3–S4 for details). Finally, 95% of all the respondents who stated they wanted to be informed about a genetic risk in the family also stated that they would have genetic testing (Item 8 and 10; Table S5).

### Whose is the moral responsibility?

Regarding the moral responsibility for informing individuals about a genetic risk within the family (Item 13), 198 of 565 (35.0%) stated that family members and HCPs share this responsibility and 148 of 565 (26.2%) believed that it lies with everyone involved, namely themselves, family members, and HCPs. Other preferences were indicated by small numbers of respondents (e.g., only HCPs *n* = 64, 11.3%; only with themselves *n* = 24, 4.2%) (Table S6).

Few participants (17 of 565, 3.0%) suggested other entities who might share the duty to inform individuals about genetic risk (Item 13a): public institutions (*n* = 11) and mass media (*n* = 6) (Table S7).

When the association of moral responsibility for informing and various participant characteristics (age, gender, parental status, genetic literacy) was evaluated using binary logistic regression models (Table S8), age was positively associated with agreement to the belief that it is a oneself responsibility (*p* = 0.016) and a HCPs’ responsibility (*p* = 0.003), and negatively associated with the belief that it is a relatives responsibility (*p* = 0.019). Conversely, female gender was also positively associated with the latter (*p* = 0.021).

### Preferences about genetic risk communication from the recipient’s perspective

**Fig. 2:**
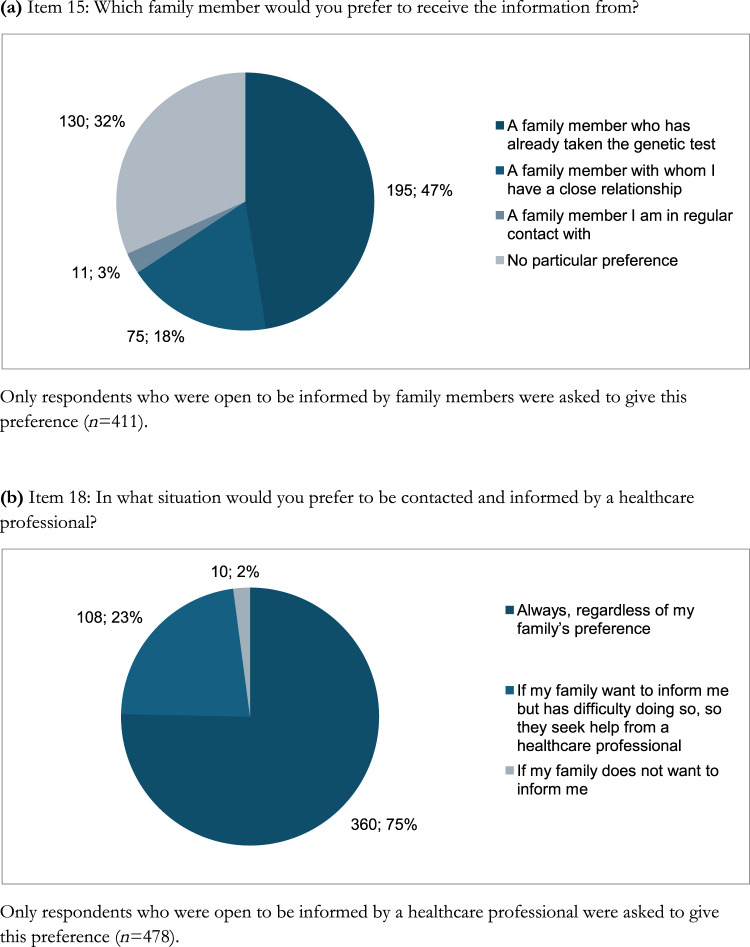
Preferences for communication from the perspective of a person who could be informed

Table 3 and Fig. 2 summarize preferences for receiving genetic risk information in the hypothetical scenarios.

**Table 3:** Preferences for communication from the perspective of a person who could receive the informatio

		Strongly disagree	Disagree	Neither agree nor disagree	Agree	Strongly agree
Items		*n* (%)	*n* (%)	*n* (%)	*n* (%)	*n* (%)
Item 14	I would prefer to be informed by a family member	10 (1.8)	30 (5.3)	114 (20.2)	203 (35.9)	208 (36.8)
Item 17	I would prefer to be contacted and informed by a healthcare professional	2 (0.4)	6 (1.1)	79 (14.0)	215 (38.1)	263 (46.5)
Item 21	I would prefer to be informed by a family member with whom I have no contact rather than not being informed at all	24 (4.2)	47 (8.3)	81 (14.3)	186 (32.9)	227 (40.2)
Item 22	I would prefer to be informed by a doctor/healthcare professional rather than not being informed at all	1 (0.2)	2 (0.4)	21 (3.7)	175 (31.0)	366 (64.8)
Item 23	I would prefer to be informed by a doctor/healthcare professional rather than by a family member with whom I have no contact	5 (0.9)	25 (4.4)	138 (24.4)	152 (26.9)	245 (43.4)
Item 24	I would prefer to be informed first by a family member with whom I have a close relationship	16 (2.8)	35 (6.2)	191 (33.8)	189 (33.5)	134 (23.7)

**Fig. 3:**
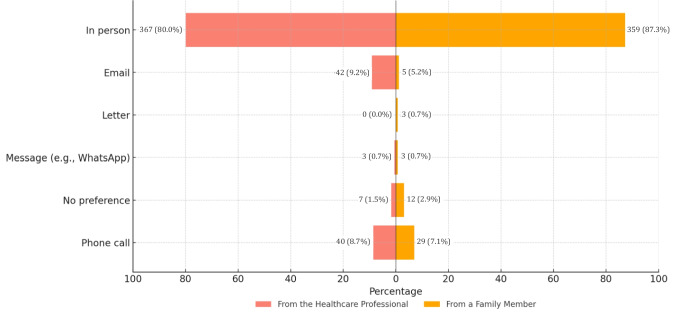
Preferred method of receiving information

Among 565 participants who declared that they would wish to be informed, 411 (72.7.%) strongly agreed or agreed that they would prefer to be informed by a family member (Item 14, Table 3). Of these, 195 (47.4%) preferred a relative who had undergone genetic testing (Item 15, Fig. 2). Most (359 of 411, 87.3%) would favor an in-person conversation (Item 16, Fig. 3).

Regarding more specific scenarios, 413 of 565 (73.1%) agreed with being informed by a family member they had no contact with rather than not being informed at all (Item 21, Table 3), and 323 of 565 (57.2%) agreed that first information should come from someone emotionally close (Item 24, Table 3).

When asked about being informed by HCPs (Item 17, Table 3), 478 of 565 (84.6%) strongly agreed or agreed. Among them, 360 (75.3%) would always prefer to be informed by a professional (Item 18, Fig. 2). In addition, 382 of 478 (79.9%) preferred being informed by a medical geneticist (Item19, Table S9). Also, 459 participants expressed their preference about the mode of information (Item 20): 367 of 459 (80.0%) would prefer an in-person consultation (Fig. 3).

A combined variable based on Items 14 and 17 showed that, among participants wanting to know at least one condition who expressed a clear preference between family members and HCPs as a source of information (330 of 565, 58.4%), 202 (61.2%) would prefer family members and 128 (38.8%) would prefer professionals (Table S10).

Regarding hypothetical scenarios, 541 of 565(95.8%) participants stated they would prefer professional disclosure over not being informed at all (Item 22, Table 3) and 397 of 565 (70.3%) agreed that they would prefer an HCP over a relative with no regular contact (Item 23, Table 3).

### Preferences about genetic risk communication from the informant’s perspective

When participants were asked to indicate which family members should be informed in a hypothetical situation (Item 26), 314 of 565 (55.6%) stated all relatives, including those with whom participants had no regular contact, 179 of 565 (31.7%) only first-degree relatives, 67 of 565 (11.9%) only those they regularly interact with and 5 of 565 (0.9%) reported none.

Most participants (510 of 565, 90.3%) agreed they would personally inform family members (Item 27), and 256 of 560 (45.7%) agreed having HCPs take on this role (Item 28) (Table 4). A combined variable based on Items 27 and 28 showed that, among the 405 of 560 (72.3%) who expressed a clear ranking, the vast majority (*n* = 359;88.6%) would prefer to inform relatives personally (Table S11). Most participants (453 of 560, 80.9%) agreed about preferring informing relatives with professional support (Item 29) (Table 4).

**Table 4:** Preferences for communication from the perspective of the informant

		Strongly disagree	Disagree	Neither agree nor disagree	Agree	Strongly agree
Items		*n* (%)	*n* (%)	*n* (%)	*n* (%)	*n* (%)
Item 27	If I was the first person in my family be diagnosed with a hereditary disease, I would personally inform my family members	2 (0.4)	8 (1.4)	45 (8.0)	172 (30.4)	338 (59.8)
Item 28*	If I were the first person in my family to be diagnosed with a hereditary disease, I would want healthcare professionals to inform my family members	19 (3.4)	82 (14.5)	203 (35.9)	158 (28.0)	98 (17.3)
Item 29*	If I were the first person in my family to be diagnosed with a hereditary disease, I would inform my family members with the help of health professionals	4 (0.7)	16 (2.8)	87 (15.4)	217 (38.4)	236 (41.8)

^*^ 5 missing values for Item 28 and Item 29

## Discussion

This study examined the hypothetical preferences of Italian laypersons towards receiving genetic risk information and sharing that information with family members, with a view to potentially informing the future design of communicative strategies aimed at improving the uptake of cascade genetic testing. Participants were presented with three hypothetical scenarios in which a close relative was found to carry a gene variant associated with a dominantly (HC, early-onset AD) or recessively (CF) heritable condition.

### Wanting to know one’s genetic risk and have genetic testing

Almost all of our participants (565/609, 93%) stated they would be interested in receiving genetic risk information about at least one of the diseases presented in the scenarios, and 95% of these also stated they would have genetic testing. These findings suggest that, if given the option, many Italian laypersons would be interested in learning about their mutational status if a hereditary condition were diagnosed in the family, and align with those reported by an increasing number of similar studies conducted in different countries (Petersen et al. 2018; Andersson et al. 2020; Marleen van den Heuvel et al. 2020; Phillips et al. 2023; Ribeiro et al. 2025). Inconsistencies emerge, however, regarding considerations of utility in decision-making linked to genetic information and testing. In some other surveys conducted among lay persons, participants expressed a significantly greater interest in being informed about genetic risks when the scenario presented was about hereditary cancer predisposition, indicating that the availability of effective treatments, surveillance, and prevention measures is critical in shaping laypersons’ preferences (Marleen van den Heuvel et al. 2020; Harrison et al. 2023; Ribeiro et al. 2025). Our results instead show that most participants (441/565, 78%) would want to be informed of their genetic risk and have testing for all the genetic conditions presented in the survey. Participants’ responses to the open question exploring why they might want to know showed that the main motivating factors were prevention for HC, reproductive responsibility for CF, and life-planning and emotional preparedness for AD, thus covering a range of elements of clinical, health-related and personal utility.

Similar results were reported by Phillips et al.(Phillips et al. 2023) in their survey on the attitudes of the Flemish population towards receiving genetic information from family members, who concluded that most participants’ willingness to be informed and get tested for an incurable disease may show that people take on a broader set of considerations than accounted for by the clinical definition of actionability (Bunnik et al. 2015; Gornick et al. 2019). In their survey of the Maltese population, Mintoff et al. also found that respondents were generally in favor of being informed of familial genetic risk and of undertaking genetic testing for conditions for which no treatment or preventive measures are available (Mintoff et al. 2024).

Our findings point to the need for further reflection on the complexity of decision-making processes around genetic testing. While population-specific sociocultural drivers underlying layperson preferences in terms of wanting to receive genetic risk information are likely to exist, they may also be challenging to identify (Mintoff et al. 2024). Furthermore, we cannot rule out that the limited detail of the scenarios provided and their hypothetical nature may have impacted our participants’ responses (Phillips et al. 2023).

### Whose is the moral responsibility?

Over a third of our study participants (198/565, 35.0%) indicated that both family members and HCPs are morally responsible for communicating genetic risk information; over a fourth (148/565 26.2%) felt that the responsibility lies with everyone involved: family members, HCPs but also themselves. Much smaller subsets indicated that only HCPs (11.3%) or participants themselves (4.2%) have the responsibility.

The concept of responsibility is central to expressing, understanding, and shaping moral perspectives on how individuals and groups handle risk. Moreover, the idea of responsible agency is used to describe and evaluate how everyday people reason morally when it comes to their obligations towards family members in sharing genetic information. (Prainsack et al. 2016).

Although the current standard practice in Italy is that probands inform relatives, with HCPs providing support through genetic counselling and, occasionally, letters to the family (Trevisan et al. 2024; Varesco et al. 2025), our findings, show that most participants viewed the responsibility to communicate genetic risk information as a shared one, suggesting that the Italian public opinion may be open to the possibility of a proactive role for HCPs in disseminating genetic risk information to family members within a collaborative approach that includes family members. This finding resonates with studies that encourage broadened communicative approaches in which interventions aimed at facilitating the sharing of genetic risk information within families take into account their relational and interdependent structure and, like our respondents, view risk communication as a shared responsibility (Zhao et al. 2022).

### Preferences about genetic risk communication

While participants generally indicated a preference to be informed of a hypothetical genetic risk by a family member, they were also open to direct communication from HCPs. Indeed, participants expressed a clear preference for receiving information from a professional rather than from an emotionally distant relative. Only a very small proportion (0.6%) indicated that they would not want to be informed if the source was an HCP. Even when family-mediated communication was favored, many respondents imagined professional involvement as a valuable complement or fallback option.

When asked to imagine themselves in the role of informant, participants were similarly supportive of HCP involvement. A collaborative model of communication was preferred, in which individuals could actively participate in disclosing risk to relatives but still rely on professional support. Previous studies have shown that the public values HCPs’ involvement in familial genetic risk communication due to their credibility and ability to deliver clear, accurate information (Frey et al. 2022; Godino et al. 2025b). Although these results reflect stated preferences within hypothetical scenarios, they suggest that, like in other countries (Menko et al. 2013; Henrikson et al. 2020; Marleen van den Heuvel et al. 2020; Tiller et al. 2023b; Lindberg et al. 2024), there may be an unmet need in Italy for more proactive HCP participation in genetic risk communication. However, support for professional involvement in our results was not an overall endorsement of HCP-led disclosure. Respondents expressed nuanced views about the appropriate timing and conditions for HCP intervention; for instance, 22.6% indicated they would only welcome direct communication from HCPs if their relatives found it difficult to convey the information themselves.

The findings of this study also indicate that while being informed is a key priority for individuals, the identity of the communicator remains significant. Many participants expressed a hypothetical preference for receiving information from emotionally close relatives or those with prior experience in genetic testing. This suggests that Italian laypersons may hold nuanced, personalized views regarding who should convey sensitive genetic information. This interpretation is consistent with previous research, which has highlighted the influence of family dynamics on the disclosure of genetic information (Wiens et al. 2013; Di Pietro et al. 2020). Such dynamics may contribute to a preference for informal communication pathways, even if these come with the emotional burden associated with sharing potentially distressing information (Leenen et al. 2016).

Moreover, over 80% of participants indicated a preference for in-person communication, regardless of whether the informant was a family member or a healthcare professional. Although these preferences were expressed in a hypothetical context, they suggest a low level of acceptability in Italy for impersonal modes of communication, including emerging AI-based tools designed to facilitate family communication of genetic risk (Nazareth et al. 2021; Siglen et al. 2022; Schmidlen et al. 2022). This is particularly relevant given the strain that in-person communication may place on clinical genetic services (Franiuk et al. 2021; Turchetti et al. 2021; Hamilton et al. 2023). To address both individual preferences and systemic resource constraints, co-designing communication strategies with members of the public and families affected by genetic conditions may offer an effective approach to balancing patient needs with healthcare system capacity.

The hypothetical preferences expressed by Italian laypersons highlight the complexity of the chain of communication involved in cascade counselling and testing, as described by families themselves (Pedrazzani et al. 2022). Like patients (Srinivasan et al. 2020), our participants seem to expect that HCPs should play an important supportive role. In the present model of care, pre- and post-test genetic counselling sessions provide key opportunities to address this complexity (Menko et al. 2013). Indeed, during these sessions, HCPs could concretely support individuals by facilitating family communication planning, offering tailored scripts, or helping them anticipate relatives’ emotional reactions.

Moreover, the common practice of drawing pedigrees during the pre-test session could be intentionally expanded beyond mapping genetic transmission. It may serve as a tool to explore relational dynamics and identify which family members are emotionally closer and potentially more suitable as communicators (Dheensa et al. 2016a; Mendes et al. 2016). These conversations could also integrate the patient’s personal values, communication preferences, and relational context, recognizing that decisions about genetic information involve more than just medical facts (Metcalfe et al. 2011; Gaff and Hodgson 2014).

While awaiting evidence-based strategies to improve communication in cascade genetic testing, Italian HCPs may begin by critically reflecting on their own present practice, particularly the narratives and assumptions they bring into counselling regarding how genetic risk information is—or should be—shared within families.

### Limitations

This study has several limitations. First, the use of an online survey may have introduced selection bias, as only social media users could learn about it and individuals with an interest in genetics or good health literacy were more likely to participate. Furthermore, all data were self-reported, making responses susceptible to social desirability bias. In addition, the demographic composition of the sample—predominantly female (68.5%) and university-educated (48.9%), with a mean age of 39.8 years—differs from the general Italian population, whose mean age is 46.6 years, with women representing about 51% and only 21.6% of adults holding a university degree (ISTAT 2023, 2024). This demographic imbalance may have influenced the findings, as younger and more educated participants are generally display by higher health literacy and a greater willingness to engage with genetic services (Ashida et al. 2011). Finally, the study relied on hypothetical scenarios, which are expected to not fully reflect real-world decision-making when individuals are personally confronted with a genetic diagnosis, as highlighted in the genetic risk communication literature (Godino et al. 2021; Henrikson et al. 2021).

## Conclusion

An increasing number of studies have shown that most people are interested in receiving genetic risk information. Consistent with this body of research, our findings, based on participants' responses to hypothetical scenarios, suggest that Italian laypersons’ desire to be informed about genetic risk is prominently linked to considerations of personal utility. Thus, we propose that bringing into focus individual needs that go beyond medical care may help improve communication about genetic risk and informed choices about genetic testing.

Our findings also indicate that many Italian laypeople view the responsibility to communicate genetic risk information as a shared one, suggesting that public opinion is open to the possibility of a proactive role for HCPs in communicating genetic risk information to family members, particularly within the frame of a collaborative effort. It is important to interpret these preferences as hypothetical, expressed in response to imagined situations, and not necessarily predictive of actual behaviors. Before interventions can be developed that incorporate these understandings in Italy, further research is needed to clarify how responsibilities can be reconceptualized and roles redefined in new engagement and communication models.

## Supplementary Information

Below is the link to the electronic supplementary material.

Supplementary file1 (DOCX 115 KB)

## Data Availability

The data that support the findings of this study are available from the corresponding author upon reasonable request.
