# Supplementary material for: “Would you want to know?” Questions of utility and responsibility in Italian laypersons’ preferences about genetic risk communication
Source: J Community Genet. 2025 Nov 18;17(1):3. doi: 10.1007/s12687-025-00837-9 (PMC12627293; doi:10.1007/s12687-025-00837-9)
Supplement: Supplementary file 1 — Supplementary file1 (DOCX 115 KB) [file 12687_2025_837_MOESM1_ESM.docx]

# Supplementary File

**File1**. Questionnaire English version

**QUESTIONNAIRE**

**Section 1: Genetic Conditions and Genetic Testing**

*Let’s start with a few general questions about hereditary diseases and genetic testing.*

1. Doctors can use genetic tests to care for their patients. Have you ever heard or read anything about this?

- Yes
- No

*If they answer “Yes”*

2. Have you ever heard or read that genetic tests can help doctors:

|  | Yes | No |
| --- | --- | --- |
| establish a person’s risk or probability of developing a specific disease? |  |  |
| decide how a disease should be managed after it is diagnosed in a person? |  |  |
| understand which drugs may or may not be effective for a person? |  |  |
| determine a person’s chance of passing on a hereditary disease to their children? |  |  |

**Section 2. Personal and Family Experience**

*Thank you for answering these general questions. Next are a few questions about your family experience. We understand that these questions may seem personal and sensitive. The reason we ask is to better understand people’s experiences about sharing genetic information within families*

3. Do you know whether there is a hereditary disease in your family?

- Yes
- No
- I do not know

4. If yes, what hereditary disease is it?

…………………………………………………………………………………………………

5. Who is affected? (multiple answers possible)

- You
- Other family member/s

6. Has a genetic test been performed for this disease in your family?

- Yes, I am the first in the family to have taken the test
- Yes, one (or more) of my family members has already taken the test, and I have too
- Yes, one (or more) of my family members has already taken the test, but I have not
- No
- I do not know

**Section 3: Hypothetical Scenarios of Hereditary Diseases**

*Thank you for answering the questions about your family experience. Now we will introduce three hypothetical situations involving* ***three different hereditary diseases****.*

*Please answer all the questions. There are no right or wrong answers. Select the option that best describes your point of view.*

Imagine that one of the following situations occurs in your family:

**Scenario 1**

One of your cousins is diagnosed with Cystic Fibrosis. Your aunt and uncle then discover that they both carry a genetic variant that causes this disease.

*Cystic Fibrosis is a genetic disease that primarily affects the lungs and the digestive system. Parents who carry one copy of a disease-causing genetic variant have no symptoms, but they can pass the variant on to their children. If both parents carry a variant, their children may develop the disease. Knowing their carrier status enables couples to make informed decisions about family planning. In Italy, about 1 in 25 people is a healthy carrier of a genetic variant that causes Cystic Fibrosis.*

**Scenario 2**

One of your aunts has ovarian cancer and has been found to carry a genetic variant that increases the risk of developing cancers such as breast, ovarian, and prostate cancer.

*People with this genetic variant are at high risk and can benefit from surveillance and prevention programs to facilitate early diagnosis and reduce their risk of developing cancer. In Italy, about 1 in 500 people has a genetic variant that increases the risk of developing these types of cancer.*

**Scenario 3**

An uncle of yours shows signs of Alzheimer's disease. Tests reveal that he carries a genetic variant that causes an early-onset form of the disease.

*Almost all individuals with this type of genetic variant develop the disease, and, currently, no preventive measures or medications can reduce the risk of this form of Alzheimer’s. This condition is very rare: the vast majority of Alzheimer's cases are* ***NOT*** *directly hereditary.*

Imagine that in these three scenarios, the uncle or aunt is your mother’s sibling, meaning you have a 25% probability (1 in 4 chance) of having inherited the genetic variant that runs on your mother’s side of the family.

**7** Would you want to know?

- Yes (*get to item 8*)
- No *(get to item 12)*

8 If your answer is yes, for which situation would you want to know?

Scenario 1 (Cystic Fibrosis) Yes/No

Scenario 2 (Cancer Predisposition) Yes/No

Scenario 3 (Alzheimer’s Disease) Yes/No

**9** Why would you want to know?

………………………………………………………………………………………………………………………………………………..……

**10** Would you want to take a genetic test to learn whether you inherited the genetic variant that runs in your family?

Scenario 1 (Cystic Fibrosis) Yes/No

Scenario 2 (Cancer Predisposition) Yes/No

Scenario 3 (Alzheimer’s Disease) Yes/No

**11** Why would you want to take the test? Why would you not? *(Get to next section*)

………………………………………………………………………………………………………………………………………………..……

**12** Why would you not want to know? (Get to items regarding Family Relationships) ………………………………………………………………………………………………………………………………………………..……

**Section 4: Moral Responsibility**

*Thank you for answering the questions about hypothetical situations. The next questions are about* ***who you think is morally responsible for informing you*** *about the genetic risk that runs in your family. Please keep in mind that when a genetic variant is found in a person with a disease, doctors usually recommend that they inform their family members, so they, in turn, can seek specialist advice to better understand their own risk. However, sharing this information with family members can be complicated or difficult. The result is that family members are often not informed.*

**13**: Who do you believe is morally responsible for informing you?

- - No one, the moral responsibility to inform myself is mine and I must be proactive about seeking the information.
  - Yes
  - No
  - I do not know

- - My family members
- Yes
- No
- I do not know

- - Doctors/Healthcare professionals
- Yes
- No
- I do not know

**13.a** Other (specifiy) ……………………………………………………………………………

**Section 5: Communication Preferences**

*Thank you for answering the questions o*n  *moral responsibility. The next questions are about* ***who you would prefer to receive the information from and how.***

*There are no right or wrong answers. Select the option that best describes your point of view.*

**14**. I would prefer to be informed by a family member.

| Strongly agree | Agree | Neither agree nor disagree | Disagree | Strongly disagree |
| --- | --- | --- | --- | --- |

*If answers “Strongly Agree” or “Agree” are given to the previous item*

**15** Which family member would you prefer to receive the information from?

- A family member who has already taken the genetic test
- A family member with whom I have a close relationship
- A family member I am in regular contact with
- No particular preference
- Other (specify) ……………………………………………………………………….

**16** How would you prefer to receive the information from your family member?

- In person
- By phone call
- By letter
- By email
- By text message (eg. WhatsApp)
- Other (specify)…………………………………………………………………

**17**. I would prefer to be contacted and informed by a healthcare professional

| Strongly agree | Agree | Neither agree nor disagree | Disagree | Strongly disagree |
| --- | --- | --- | --- | --- |

*If answers “Strongly Agree” or “Agree” are given to the previous item*

**18** In what situation would you prefer to be contacted and informed by a healthcare professional?

- Always, regardless of my family’s preference
- If my family want to inform me but has difficulty doing so, so they seek help from a healthcare professional
- If my family does not want to inform me
- Other (specify) ………………………………………………………………….

**19**. Which healthcare service or professional would you prefer to be informed by? (multiple answers possible)

- The genetics service that counseled/tested my family members (medical geneticist)
- The genetics service that counseled/tested my family members (genetic nurse)
- The genetics service closest to where I live (medical geneticist)
- The genetics service closest to where I live (genetic nurse)
- My family doctor
- My family nurse
- The public health-prevention service
- No particular preference
- Other (specify) ……………………………………………………………….

**20** How would you prefer to receive the information from a healthcare professional?

- In person
- By phone call
- By letter
- By email
- By text message (eg. WhatsApp)
- Other (specify)…………………………………………………………………

**21** I would prefer to be informed by a family member with whom I have no contact rather than not being informed at all

| Strongly agree | Agree | Neither agree nor disagree | Disagree | Strongly disagree |
| --- | --- | --- | --- | --- |

**22** I would prefer to be informed by a doctor/healthcare professional rather than not being informed at all.

| Strongly agree | Agree | Neither agree nor disagree | Disagree | Strongly disagree |
| --- | --- | --- | --- | --- |

**23** I would prefer to be informed by a doctor/healthcare professional rather than by a family member with whom I have no contact.

| Strongly agree | Agree | Neither agree nor disagree | Disagree | Strongly disagree |
| --- | --- | --- | --- | --- |

**24** I would prefer that the first communication comes by a family member with whom I have a close relationship.

| Strongly agree | Agree | Neither agree nor disagree | Disagree | Strongly disagree |
| --- | --- | --- | --- | --- |

**25** If you want to add any comments or share any information that you consider relevant, please use the space below:

………………………………………………………………………………………………………………………………………………..……

**Section 6: Disclosure of Personal Diagnosis**

Thank you for answering the questions about who you would prefer to be informed by. Now imagine that you are the first person in your family to have the genetic test. The following are questions about **how you would or would not want to share tha**t **information with your family.**

*There are no right or wrong answers. Select the option that best describes your point of view.*

**26**. If I were the first person in my family to be diagnosed with a hereditary disease, I would want the following family members to be informed:

- Only close family members (parents, siblings, children) (*Get to next item)*
- Only family members with whom I have regular contact *(Get to next item)*
- All my family members, even those I don’t have regular contact with (*Get to next item)*
- None of my family members *(Get to item 30)*

**27** If I was the first person in my family be diagnosed with a hereditary disease, I would personally inform my family members

| Strongly agree | Agree | Neither agree nor disagree | Disagree | Strongly disagree |
| --- | --- | --- | --- | --- |

**28** If I were the first person in my family to be diagnosed with a hereditary disease, I would want healthcare professionals to inform my family members

| Strongly agree | Agree | Neither agree nor disagree | Disagree | Strongly disagree |
| --- | --- | --- | --- | --- |

**29** If I were the first person in my family to be diagnosed with a hereditary disease, I would inform my family members with the help of healthcare professionals

| Strongly agree | Agree | Neither agree nor disagree | Disagree | Strongly disagree |
| --- | --- | --- | --- | --- |

**30** Why would you not want your family members to be informed?

………………………………………………………………………………………………………………………………………………..……

**31** If you would like to add any comments or share any information you find relevant, please use the space below

………………………………………………………………………………………………………………………………………………..……

**Section 7: Family Relationships**

Thank you for answering the queries regarding who would you rather inform in your family.

Now we would like you to tell us something about how you see your family in this moment. So we ask you YOUR personal point of view about your biological family *(those* relatives that are genetically related to you like parents, siblings and children)

For each question answer matching the proposition that better describes your family, from 1 (Very well) to 5 (Not at all)

Do not dwell too much on each question, but answers to all of them anyway.

**32.**

|  | Very well | Well | Partly | Not well | Not at all |
| --- | --- | --- | --- | --- | --- |
| In my family we talk to each other about things which matter to us |  |  |  |  |  |
| People often don’t tell each other the truth in my family |  |  |  |  |  |
| Each of us gets listened to in our family |  |  |  |  |  |
| It feels risky to disagree in our family |  |  |  |  |  |
| We find it hard to deal with everyday problems |  |  |  |  |  |
| We trust each other |  |  |  |  |  |
| It feels miserable in our family |  |  |  |  |  |
| When people in my family get angry they ignore each other on purpose |  |  |  |  |  |
| We seem to go from one crisis to another in my family |  |  |  |  |  |
| When one of us is upset they get looked after within the family |  |  |  |  |  |
| Things always seem to go wrong for my family |  |  |  |  |  |
| People in the family are nasty to each other |  |  |  |  |  |
| People in my family interfere too much in each other’s lives |  |  |  |  |  |
| In my family we blame each other when things go wrong |  |  |  |  |  |
| We are good at finding new ways to deal with things that are difficult |  |  |  |  |  |

**33** If you would like to add any comments or share any information you find relevant, please use the space below

………………………………………………………………………………………………………………………………………………..……

**Section 8: Socio-Demographic Characteristics**

We ask you finally to answer to some queries regarding socio-demographic aspects

**How old are you?**

_____ (number)

**Gender**

- Male
- Female
- I prefer to not declare it

**What is your current marital status?**

- Single
- Married
- Cohabiting
- Divorced

**Do you have children?**

- Yes
- No

**Do you plan to have children or to increase the family with others?**

- Yes
- No
- Maybe
- I do not know

**Which is your highest level of education?**

- Primary School Diploma
- Middle School Diploma
- High School Diploma
- Bachelor’s Degree
- Postgraduate Specialization
- Doctorate (PhD)

**What is your current employment status?**

- Paid employment
- Voluntary work
- Student
- Household responsibilities
- Retired
- Other (specify) ………………………………….


   **If you have a paid employment, do you work as a healthcare professional?**

- Yes
- No

  If “Yes” to the previous question

**Which one is your profession?**

- Healthcare assistant
- Biologist
- Chemist
- Dietitian
- Professional educator
- Pharmacist
- Physician
- Physiotherapist
- Dental hygienist
- Nurse
- Pediatric Nurse
- Speech therapist
- Medical doctor
- Odontologist
- Orthotic and ophthalmologic assistant
- Midwife
- Podiatrist
- Psychologist
- Audiometric technician
- Acoustic-aid technician
- Cardiovascular perfusionist
- Environment and Workplace Prevention Technician
- Neurophysiopathology technician
- Orthopaedic technician
- Technical of the psychiatric rehabilitation
- Biomedical laboratory technician
- X-ray technician
- Neuro and Psychomotor Therapist of Developmental Age
- Occupational Therapist
- Veterinarian

**How do you approach to religion?**

- Practicing Believer *(which religion? Next question)*
- Non-Practicing Believer *(which religion? Next question)*
- Non Believer *(go to question regarding “Residence area”)*

Other (specify): ____________________


**How would you define yourself:**

- Catholic christian
- Muslim
- Other (specify): ____________________


   **What is your current area of residence?**
   □ Abruzzo
   □ Basilicata
   □ Calabria
   □ Campania
   □ Emilia-Romagna
   □ Friuli-Venezia Giulia
   □ Latium
   □ Liguria
   □ Lombardy

   □ Marche
   □ Molise
   □ Piedmont
   □ Apulia
   □ Sardinia
   □ Sicily
   □ Tuscany
   □ Trentino South Tyrol
   □ Umbria
   □ Aosta Valley
   □ Veneto
   □ Residency outside Italy


  *Thank you for responding to our questionnaire!*

**Table S1.** Questions regarding family relationship (Score-15)

|  | **Answers** | | | | | | | | | |
| --- | --- | --- | --- | --- | --- | --- | --- | --- | --- | --- |
|  | ***Very well*** | | ***Well*** | | ***Partly*** | | ***Not well*** | | ***Not at all*** | |
| **Questions** | **n** | **%** | **n** | **%** | **n** | **%** | **n** | **%** | **n** | **%** |
| In my family we talk to each other about things which matter to us | 248 | 40.7 | 208 | 34.2 | 104 | 17.1 | 41 | 6.7 | 8 | 1.3 |
| People often don’t tell each other the truth in my family | 21 | 3.4 | 52 | 8.5 | 85 | 14.0 | 235 | 38.6 | 216 | 35.5 |
| Each of us gets listened to in our family | 175 | 28.7 | 189 | 31.0 | 186 | 30.5 | 47 | 7.7 | 12 | 2.0 |
| It feels risky to disagree in our family | 78 | 12.8 | 129 | 21.2 | 181 | 29.7 | 117 | 19.2 | 104 | 17.1 |
| We find it hard to deal with everyday problems | 16 | 2.6 | 49 | 8.0 | 97 | 15.9 | 252 | 41.4 | 195 | 32.0 |
| We trust each other | 260 | 42.7 | 182 | 29.9 | 131 | 21.5 | 28 | 4.6 | 8 | 1.3 |
| It feels miserable in our family | 27 | 4.4 | 48 | 7.9 | 73 | 12.0 | 238 | 39.1 | 223 | 36.6 |
| When people in my family get angry they ignore each other on purpose | 14 | 2.3 | 45 | 7.4 | 109 | 17.9 | 232 | 38.1 | 209 | 34.3 |
| We seem to go from one crisis to another in my family | 15 | 2.5 | 27 | 4.4 | 84 | 13.8 | 217 | 35.6 | 266 | 43.7 |
| When one of us is upset they get looked after within the family | 161 | 26.4 | 184 | 30.2 | 163 | 26.8 | 79 | 13.0 | 22 | 3.6 |
| Things always seem to go wrong for my family | 10 | 1.6 | 21 | 3.4 | 77 | 12.6 | 214 | 35.1 | 287 | 47.1 |
| People in the family are nasty to each other | 6 | 1.0 | 23 | 3.8 | 25 | 4.1 | 118 | 19.4 | 437 | 71.8 |
| People in my family interfere too much in each other’s lives | 15 | 2.5 | 28 | 4.6 | 88 | 14.4 | 215 | 35.3 | 263 | 43.2 |
| In my family we blame each other when things go wrong | 9 | 1.5 | 20 | 3.3 | 67 | 11.0 | 199 | 32.7 | 314 | 51.6 |
| We are good at finding new ways to deal with things that are difficult | 111 | 18.2 | 186 | 30.5 | 228 | 37.4 | 64 | 10.5 | 20 | 3.3 |

**Table S2.** The four genetic literacy-based statments

| **Genetic Literacy** | No | | Yes | |
| --- | --- | --- | --- | --- |
|  | n | % | n | % |
| *Determining the risk or probability of developing a specific disease* | 250 | 41.1 | 359 | 58.9 |
| *Determining how a disease should be managed following diagnosis* | 384 | 63.1 | 225 | 36.9 |
| *Determining which medications may or may not be effective for an individual* | 375 | 61.6 | 234 | 38.4 |
| *Determining the probability of transmitting a hereditary disease to offspring* | 249 | 40.9 | 360 | 59.1 |

**Table S3.** Qualitative Analysis– Comparison Between Participants Choosing One or Two Conditions and Those Choosing All Three

| **Themes** | **Participants who chose one or two conditions**  **(n = 124)** | **Participants who wanted to be informed about all three conditions**  **(n = 441)** |
| --- | --- | --- |
| Prevention | Strongly present in the cancer scenario, less so in others. Often focused on concrete and immediate actions.  *“To undergo more frequent check-ups for prevention”*  *“To start a thorough screening plan”* | Very common and often accompanied by reflections on lifestyle, screening, and clinical anticipation.  *“I would undergo regular screening to help monitor the situation”*  *“To have more targeted and frequent check-ups”* |
| Awareness | Less frequently expressed and often vague. Sometimes emerges through general statements such as “just to know”.  *“To be aware of it”*  *“To explore more deeply”* | A central theme, often tied to the desire to inform family members or to feel safer and more prepared.  *“To be informed about my family’s diseases”*  *“To know the exact probability of being affected”* |
| Control / Managing uncertainty | Mostly reported in the Alzheimer’s scenario, often with emotional overtones or fear.  *“To know how many years I can hope to live”*  *“Because I’m afraid of this disease”* | More widespread and articulated.  *“To prepare myself for what might happen”*  *“To have a clearer view of the future and make informed choices”* |
| Family responsibility | Clearly expressed in the reproductive and cancer scenarios.  *“To make more informed decisions about my life and those around me”*  *“Out of responsibility toward my family members”* | Also mentioned in the Alzheimer’s scenario: protecting children, planning inheritance, sharing information with relatives.  *“To protect my children”*  *“To have my family’s future financially and emotionally organized”* |
| Psychological preparation | Often expressed in emotional terms, not always supported by practical considerations.  *“To prepare myself and my loved ones in advance”*  *“Fear”* | Accompanied by references to concrete strategies, life planning, and future care.  *“To face a possible diagnosis with clarity”*  *“To plan for my future, including financial and family aspects”* |

**Table S4.** Qualitative Analysis– Comparison Between the two groups (Participants Choosing One or Two Conditions and Those Choosing All Three) by clinical scenario

| **Scenario** | **Participants who chose one or two conditions**  **(n = 124)** | **Participants who wanted to be informed about all three conditions**  **(n = 441)** |
| --- | --- | --- |
| Cystic Fibrosis | Motivations are mainly reproductive (e.g., avoiding disease transmission to children, use of assisted reproduction), with some references to family planning and early surveillance.   Main theme: Reproductive responsibility  41.9% indicated a desire to know about CF. | Motivations remain centered on reproduction but are more articulated, including the desire to inform other family members, plan for the future, and explore reproductive strategies.   Main theme: Reproductive responsibility + informed reproductive choices |
| Hereditary Cancer | This is the most activating scenario: prevention is the dominant motivation, often accompanied by family history and a sense of responsibility toward oneself and others.   Main theme: Active clinical prevention  90.3% indicated a desire to know about HBOC. | The same motivations emerge, but in a broader and more internalized manner: prevention, lifestyle changes, regular screenings. Family history is mentioned more often as a background factor.   Main theme: Integrated and proactive prevention |
| Early-Onset Alzheimer’s | Motivations are less clinical and more emotional: the need for psychological preparation and the management of fear and uncertainty. Few references to concrete action plans.   Main theme: Managing uncertainty and the future  33.9% indicated a desire to know about Alzheimer’s risk. | Existential motivations also emerge here, but in a more structured and conscious way: some participants mention financial planning, caring for loved ones, and making life plans.   Main theme: Family preparation and long-term planning |

**Table S5 Comparison between willingness to know about genetic risk and willingness to undergo genetic testing**

|  | | **Willingness to undergo genetic testing for** | |
| --- | --- | --- | --- |
| **Willingness to know** | | *Cystic Fibrosis* | |
|  |  | **No**  **n (%)** | **Yes**  **n (%)** |
| *Cystic Fibrosis* | *No* | 61 (84.7) | 11 (15.3) |
|  | *Yes* | 26 (5.3) | 467 (94.7) |
|  |  | *Hereditary Cancer* | |
| *Hereditary Cancer* | *No* | 12 (100.0) | 0 (0.0) |
|  | *Yes* | 19 (3.4) | 534 (96.6) |
|  |  | *Early-Onset Alzheimer's disease* | |
| *Early-Onset Alzheimer's disease* | *No* | 72 (87.8) | 10 (12.2) |
|  | *Yes* | 23 (4.8) | 460 (95.2) |

# Table S6. Comparison between participants’ views on moral responsibility for communicating genetic risk and willingness to be informed

|  |  | **Total (N=565)** |
| --- | --- | --- |
| **Item 13.** In your opinion, who has the moral responsibility to inform you? | |  |
|  |  | **n (%)** |
| *All (self, relatives, healthcare professionals)* | | 148 (26.2) |
| *Only self* | | 24 (4.2) |
| *Only relatives* | | 45 (8.0) |
| *Only healthcare professionals* | | 64 (11.3) |
| *Both relatives and healthcare professionals* | | 198 (35.0) |
| *Both self and relatives* | | 32 (5.7) |
| *Both self and healthcare professionals* | | 41 (7.3) |
| *No one/Not known* | | 13 (2.3) |

**Table S7. Qualitative analysis of additional comments given on the theme of moral responsibility (item 13a)**

| **Category** | **Subcategory** | **Example from participant responses** |
| --- | --- | --- |
| Public institutions | Ministry of Health | *The Ministry of Health should carry out awareness campaigns.* |
|  | Regional health authorities | *The region, the pediatrician, and the family doctor, all the way up to the Ministry of Health, should provide information through public campaigns.* |
|  | The State / National Health System (SSN) | *The State, because prevention is always better than cure.*  *The SSN should be automated to provide population-wide checks to maximize prevention.* |
|  | Public information services | *Public information services should implement widespread prevention and information campaigns.* |
|  | General public authorities | *There should be more public information.*  *Public institutions with awareness campaigns.* |
| Mass media | Television and newspapers | *Television news should inform the public.*  *Qualified mass media.*  *Campaigns through media outlets.* |
|  | Social media and digital platforms | *Media channels such as social networks or television.*  *Newspapers, social.*  *Targeted communications through press, web, flyers, broadcasts.* |
| Educational settings | Schools | *In my opinion, this kind of information should be shared by as many people as possible. In particular, a ‘sensitization’ program in schools would be appropriate.* |

**Table S8.** Binary Logistic Regression Analyses on Perceived Moral Responsibility

|  | **Self**  **(n=555)** | | **Relatives**  **(n=555)** | | **Professionals**  **(n=555)** | |
| --- | --- | --- | --- | --- | --- | --- |
| Predictors | OR [95% CI] | *p-value* | OR [95% CI] | *p-value* | OR [95% CI] | *p-value* |
| Age | 1.02 [1.00–1.03] | 0.016 | 0.98 [0.97–0.99] | 0.019 | 1.03 [1.01–1.05] | 0.003 |
| Gender | 0.77 [0.53–1.12] | 0.150 | 1.63 [1.04–2.47] | 0.021 | 1.31 [0.80–2.00] | 0.300 |
| Genetic Literacy | 1.01 [0.92–1.11] | 0.867 | 1.00 [0.90–1.12] | 0.971 | 0.96 [0.85–1.08] | 0.519 |
| Having children | 0.95 [0.61–1.48] | 0.818 | 0.93 [0.56–1.53] | 0.769 | 0.58 [0.33–1.03] | 0.062 |

Note. OR = Odds Ratio; CI = Confidence Interval. Self = 'I have the moral responsibility'; Relatives = 'My family members have the moral responsibility'; Professionals = 'Healthcare professionals have the moral responsibility'. Gender is coded as 0 = male (reference), 1 = female. Having children is coded as 0 = no (reference), 1 = yes; Knowing all conditions is coded as 0 = Knowing one or two conditions (reference), 1 = Knowing all three conditions

**Table S9.** Item18 - Preferred healthcare source for receiving genetic information (multiple choices allowed)

|  | **Yes** | | **No** | |
| --- | --- | --- | --- | --- |
|  | n | % | n | % |
| The genetics service that counseled/tested my family members (medical geneticist) | 277 | 57.9 | 201 | 42.1 |
| The genetics service that counseled/tested my family members (genetic nurse) | 100 | 20.9 | 378 | 79.1 |
| The genetics service closest to where I live (medical geneticist) | 105 | 22.0 | 373 | 78.0 |
| The genetics service closest to where I live (genetic nurse) | 52 | 10.9 | 426 | 89.1 |
| My family doctor | 182 | 38.1 | 296 | 61.9 |
| My family nurse | 33 | 7.4 | 445 | 93.1 |
| The public health-prevention service | 110 | 23.0 | 368 | 77.0 |
| No particular preference | 151 | 31.6 | 327 | 68.4 |

**Table S10.** Ranking of Respondents’ Preferences for Receiving Genetic Risk Information: Family Members versus Healthcare Professionals

| ***Participants preferred to receive genetic risk information from...*** | ***Total (N=565)*** | |
| --- | --- | --- |
|  | ***n*** | ***%*** |
| Family members | 202 | 35.8 |
| Healthcare professionals | 128 | 22.7 |
| Equally from both (including don’t know) | 16 | 2.8 |
| Disagree with both | 219 | 38.8 |

**Table S11.** Ranking of Respondents’ Preferences for Informing their relatives about own genetic Risk

| ***If I were the first person in my family to be diagnosed with a hereditary disease...*** | ***Total (N=560)**** | |
| --- | --- | --- |
|  | ***n*** | ***%*** |
| I would personally inform my family members | 359 | 64.1 |
| I would want healthcare professionals to inform relatives | 46 | 8.2 |
| Equally from family members or healthcare professionals both (including don’t know) | 153 | 27.3 |
| Disagree with both | 2 | 0.4 |

* 5 missing
